# Characterization of the Key Aroma Compounds in Proso Millet Wine Using Headspace Solid-Phase Microextraction and Gas Chromatography-Mass Spectrometry

**DOI:** 10.3390/molecules23020462

**Published:** 2018-02-20

**Authors:** Jingke Liu, Wei Zhao, Shaohui Li, Aixia Zhang, Yuzong Zhang, Songyan Liu

**Affiliations:** 1 Institute Millet Crops of Heibei Academy of Agriculture and Forestry, Shijiazhuang 050035, China; zhaoweipg@163.com (W.Z.); lishaohui007@163.com (S.L.); zhangaixia1977@126.com (A.Z.); zyz56@163.com (Y.Z.); 2 National Millet Improvement Center of China, Shijiazhuang 050035, China; 3 Minor Cereal Crops Research Laboratory of Hebei Province, Shijiazhuang 050035, China; 4 Shijiazhuang Livestock Products Quality Inspection & Supervision Center, Shijiazhuang 050041, China; lsyan@163.com

**Keywords:** aroma compounds, proso millet wine, headspace solid-phase microextraction, gas chromatography-mass spectrometry

## Abstract

The volatile compounds in proso millet wine were extracted by headspace solid-phase microextraction (85 μm polyacrylate (PA), 100 μm polydimethylsiloxane (PDMS), 75 μm Carboxen (CAR)/PDMS, and 50/30 μm divinylbenzene (DVB)/CAR/PDMS fibers), and analyzed using gas chromatography-mass spectrometry; the odor characteristics and intensities were analyzed by the odor activity value (OAV). Different sample preparation factors were used to optimize this method: sample volume, extraction time, extraction temperature, and NaCl addition. A total of 64 volatile compounds were identified from the wine sample, including 14 esters, seven alcohols, five aldehydes, five ketones, 12 benzene derivatives, 12 hydrocarbons, two terpenes, three phenols, two acids, and two heterocycles. Ethyl benzeneacetate, phenylethyl alcohol, and benzaldehyde were the main volatile compounds found in the samples. According to their OAVs, 14 volatile compounds were determined to be odor-active compounds (OAV > 1), and benzaldehyde, benzeneacetaldehyde, 1-methyl-naphthalene, 2-methyl-naphthalene, and biphenyl were the prominent odor-active compounds (OAV > 50), having a high OAV. Principal component analysis (PCA) showed the difference of distribution of the 64 volatile compounds and 14 odor-active compounds with four solid-phase microextraction (SPME) fibers.

## 1. Introduction

Proso millet (*Panicum miliaceum* L.) is an important grain crop grown in arid areas. It plays a very important role in the agriculture and food industries in many developing countries because of its capacity to grow under adverse heat and limited rainfall conditions [1]. It has a fairly short growing season and will mature into a grain crop 60–75 days after seeding. It can serve as an emergency cash crop or a catch crop after others have failed [2]. Besides having agronomic advantages, proso millet has better amino acid composition and high nutritive value, which is comparable to that of major cereals such as wheat, corn, and rice [3]. It has significant food processing potential. Proso millet is used in the production of pasta [3], weaning mix [4], and breads and cookies [5]. In China, yellow wine is an important product that is made from proso millet and is a traditional fermented alcoholic drink, especially in Shaanxi, Gansu, and Shanxi provinces, where it has a long history.

Proso millet wine is a type of traditional Chinese yellow wine. One of the most important quality markers for yellow wine is the aroma. The aroma of wines is directly related to the degree of consumer preference. Aroma is considered of primary importance in that superior scent increases consumer satisfaction, overall acceptability, and the probability of repeated purchase [6]. Both wines from proso millet and rice belong to cereal fermented wine with similar processing technology. Published research on the aroma of yellow wine has mainly focused on rice wine. The key characteristic aroma of rice wine is typically described as having “caramel”, “herbal”, “smoky”, “yeasty”, “moldy”, “Qu-like aroma”, and “honey aroma” characteristics [6]. In the study of rice wine, esters, alcohols, sulfur compounds, and carbonyl compounds were reported to contribute to the aroma of rice wine, especially 3-hydroxy-4,5-dimethyl-2(5H)-fura none, which was identified as a burnt flavoring compound in rice wine aroma [7,8]. Other compounds, such as β-phenylethyl alcohol, vanillin, benzaldehyde, and so on, also had important contributions to the aroma of rice wine [6]. However, these studies mainly investigated the aroma of rice wine; thus, the volatile compounds and odor-contributing compounds of proso millet wine are still not well defined.

Solid-phase microextraction (SPME) has been established as an analyte sampling/enrichment approach for trace compound analysis in various sample matrices. This technique has been applied to the analysis of volatile compounds in alcoholic beverages such as rice wine [9], Chinese liquor [10], beer [11], cider [12], and cold-hardy wine [13] because of its ease of use, good reproducibility, and lack of a need for either large samples or solvents of any kind [14]. Fiber coating is the key to SPME because different fibers have different selection and extractive efficiency of volatile compounds. Different fibers were used in SPME to analyze the volatile food compounds of raspberry [15], citrus [16], horsemeat [17], duck [18], and bean curd [19], giving full play to the efficiency of volatile compounds complemented by different fibers. Hence, an overall comprehension of the volatile compounds of these food products could be reached. In addition, the composition of food volatile compounds is complicated. The limited numbers of compounds, which contribute to the odor, are called key odor-active components. Odor activity value (OAV) is an important method that is used to select key odor components by using the ratio of the concentration of a certain odor to its threshold value to determine the contribution of the odor component to food flavor. The contribution of volatiles to the final aroma depends on those odorants with OAV > 1. Thus, OAV was widely introduced for choosing impactful odorants in alcoholic beverages [20], and successfully defined the odor characteristics of this kind beverages, which provides a theoretical basis for industrial production control and consumer drinking.

The objective of this study was to test different fibers using headspace solid-phase microextraction (HS-SPME) as well as to use gas chromatography-mass spectrometry (GC-MS) to analyze the volatile compounds in proso millet wine and evaluate the contributions of specific volatile compounds on the aroma of the overall sample.

## 2. Results

### 2.1. Optimization of the Extraction Method

**Figure 1:**
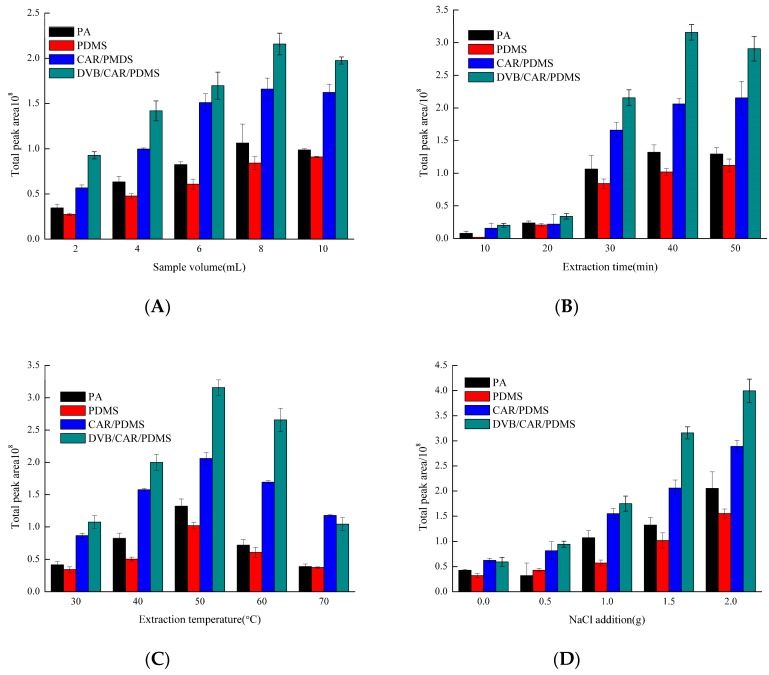
Effects of (**A**) sample volume, (**B**) extraction times, (**C**) extraction temperatures, and (**D**) NaCl addition on the extraction effects. (**A**) extraction time: 40 min, extraction temperature: 40 °C, NaCl: 1 g; (**B**) sample volume: 8 mL, extraction temperature: 40 °C, NaCl: 1 g; (**C**) sample volume: 8 mL, extraction time: 40 min, NaCl: 1 g; (**D**) sample volume: 8 mL, extraction time: 40 min, extraction temperature: 60 °C.

There are many factors that can have a direct impact during the HS-SPME process. These include the sample volume, extraction time, extraction temperature, and NaCl amount. From Figure 1, the different fibers had a similar adsorption trend under different extraction conditions. The difference is mainly concentrated on adsorption capacity; the highest to lowest adsorption capacity was from divinylbenzene (DVB)/Carboxen (CAR)/polydimethylsiloxane (PDMS), CAR/PDMS, polyacrylate (PA), then PDMS.

#### 2.1.1. Sample Volume

Volumes of 2, 4, 6, 8, and 10 mL were selected to determine how the sample volume affects total peak area (Figure 1A). Results showed that total peak area with sample volume of 6, 8, and 10 mL were higher than those of 2 and 4 mL. In consideration of extraction efficiency, 8 mL served as the optimal sample volume. Because the HS-SPME mechanism is based on the equilibrium of analytes among three phases (polymeric coating, headspace, and sample), sample volume directly affects two phases of the headspace and sample, which in turn influences extraction efficiency [21]. The volume of analyte removed by the fiber is proportional to the compound concentration in the sample volume. Along with the increase in the sample volume, the concentration of volatile compounds increases in the headspace. When volatile compounds were in equilibrium concentration with the fiber, no clear change in absorption efficiency was observed. The equilibrium might have been reached for some compounds (typically those with low MW), it was not likely to reach for heavier MW compounds.

#### 2.1.2. Extraction Time

Extraction time also affects extraction efficiency. The proso millet wine samples were extracted for 10, 20, 30, 40, and 50 min, respectively (Figure 1B). Results showed that the total peak area increased with extraction time extension. Little change was found when it was prolonged from 40 to 50 min. Therefore, 40 min was chosen as the optimal extraction time. Extraction time is the time required for an analyte to reach equilibrium between the sample matrix and the stationary phase [19], which is one of the most important parameters in the HS-SPME process because it influences the equilibrium of the analytes between the headspace and the fiber coating.

#### 2.1.3. Extraction Temperature

Different extracting temperatures (30, 40, 50, 60, and 70 °C) were evaluated in the HS-SPME parameter screening experiment (Figure 1C). The results showed that the peak area of volatile compounds increased first and then decreased, then reached the highest when extracting temperature was 50 °C. Thus, 50 °C was found to be the suitable extraction temperature. In general, heat provides energy for analyte molecules to overcome energy barriers that tie it to the matrix, thus facilitating the release of analytes into the headspace. However, it can adversely affect adsorption of analytes by coating due to the decrease in partition coefficients [22].

#### 2.1.4. NaCl Addition

NaCl levels of 0, 0.5, 1.0, 1.5, and 2.0 g were selected to test the effect concentration of NaCl on the total peak area. The total peak area increased as NaCl addition increased; 2.0 g was the most suitable NaCl level (Figure 1D). In the SPME procedure, the ‘salting out effect’ was used to modify the matrix through the addition of salts such as NaCl to increase the ionic strength of the water and therefore decrease the solubility of analytes and increase the release of analytes into the headspace, thereby contributing to enhanced adsorption onto the fiber [22].

Then, 8 mL of the sample was placed in a 15 mL headspace vial with 2.0 g of NaCl. Extraction occurred at 50 °C for 40 min for SPME fibers with different coatings.

### 2.2. Volatile Compounds in Proso Millet Wine

Table 1 shows volatile compounds of proso millet wine and their OAVs. A total of 64 volatile compounds were identified from the sample using SPME with different coatings, including 14 esters, seven alcohols, five aldehydes, five ketones, 12 benzene derivatives, 12 hydrocarbons, two terpenes, three phenols, two acids, and two heterocycles. Forty, 37, 42, and 44 volatile compounds, which have a total concentration of 34067.24 ± 2037.95 μg/L, 26336.79 ± 763.85 μg/L, 56864.85 ± 2010.45 μg/L, and 64667.24 ± 2002.81 μg/L, respectively, were detected in PA, PDMS, CAR/PDMS, and DVB/CAR/PDMS, respectively. Among the 64 quantitated compounds, in four fiber coatings, 14 compounds reached concentrations higher than their odor threshold. Six, 8, 13, and 12 aroma-active compounds were identified in PA, PDMS, CAR/PDMS, and DVB/CAR/PDMS, respectively. The volatile compounds and their OAVs detected from the SPME with different fibers were noticeably different. The volatile components presented in the sample were overall understood using SPME with different fibers.

#### 2.2.1. Esters

Esters were the largest group in terms of the number of aroma compounds identified in the sample. A total of 14 esters were detected in samples, and the subtotal concentration of esters varied from 5192.56 to 11482.29 μg/L in four different SPME fibers. The formation of esters was significantly influenced by the SPME fibers. There were 10, 9, 11, and 12 esters detected in PA, PDMS, CAR/PDMS, and DVB/CAR/PDMS, respectively. Esters showed a higher degree of similarity using different fibers. Among them, isoamyl lactate, ethyl benzoate, ethyl benzeneacetate, ethyl 3-phenylpropanoate, dimethyl phthalate, and ethyl hexadecanoate were detected in all fibers. Most of these esters were the main ester which was detected in high concentration, such as ethyl benzeneacetate and dimethyl phthalate, which exceeded 1000 μg/L in four fibers. Ethyl hexadecanoate exceeded 1000 μg/L in CAR/PDMS and DVB/CAR/PDMS fibers. Dimethyl phthalate is usually a contaminant coming from plastic containers where samples were stored, and in wine [26]. Esters also represented the largest group in rice wine. Actually, all ethyl esters have been detected in rice wine except ethyl dodecanoate [6,9]. Esters are mainly produced by the esterification of alcohols with fatty acids or the biosynthesis of alcohol acetyl transferase using higher alcohols and acetyl-CoA as substrates [27]. Esters can impact the quality of alcohol beverages for desirable fruity and flowery flavors [23]. Among 14 esters, only the OAVs of four esters were greater than 1; these four could have a significant impact on sample aroma. Ethyl 3-phenylpropanoate, which had an OAV in the SMPE of different fibers that exceeded 5, contributed fruity and sweet notes to wine. The OAV of ethyl dodecanoate and ethyl hexadecanoate, in CAR/PDMS and DVB/CAR/PDMS, were greater than 1 and ranged from 1 to 2; they contributed to the aroma of the sample. Ethyl benzoate and ethyl decanoate, which had OAVs just greater than 1 but only in CAR/PDMS, also presented as being fruity and sweet.

#### 2.2.2. Alcohols

Seven alcohols were detected in samples containing different fibers. Six, 3, 3, and 6 alcohols, with subtotal concentrations of 14982.57 ± 1464.48 μg/L, 3546.66 ± 339.05 μg/L, 7360.57 ± 800.93 μg/L, and 13403.63 ± 1335.52 μg/L, respectively, were detected in PA, PDMS, CAR/PDMS, and DVB/CAR/PDMS, respectively. PA and DVB/CAR/PDMS appeared to have good adsorbing effect for alcohols. Phenylethyl alcohol and 4-methyl-1-(1-methylethyl)-3-cyclohexen-1-ol were detected in all fibers. However, 2-furanmethanol and 4-trimethyl-3-cyclohexene-1-methanol only were detected in DVB/CAR/PDMS and PA, respectively. Phenylethyl alcohol, which had a concentration ranging from 2996.79 μg/L to 12826.54 μg/L in four fibers, was the most abundant alcohol in each fiber. Alcohols could be formed during the fermentation process, i.e., under aerobic conditions from sugar and under anaerobic conditions from amino acids [27]. Since the raw proso millet is a rich source of amino acids, alcohols can be converted from amino acids by the Ehrlich metabolic pathway [28]. Phenylethyl alcohol was the most abundant element in rice wine, which was an important quality standard in Chinese rice wine [6,9]. Alcohols usually contribute to floral and fruity notes in the sample. Among four aroma characteristic alcohols, only OAV of phenylethyl alcohol was greater than 1, ranging from 3.3 in PDMS fiber to 14.3 in PA fiber. Phenylethyl alcohol is produced from the conversion of L-phenylalanine, present in the fermentation medium, by yeast during amino acid metabolism or from sugar by de novo synthesis [29]. Phenylethyl alcohol should be noted, which is a compound with a floral and fruity like flavor, which had high content and high OAV in sample. It has been detected as an important odor component in other cereal wines such as rice wine [9] and in foxtail millet sake [30], and is regarded as an important quality standard.

#### 2.2.3. Carbonyl Compounds

Carbonyl compounds in the samples included aldehydes and ketones. In the proso millet wine liquor sample, five aldehydes and five ketones were detected using SPME with different fibers. Samples showed relatively high concentration of aldehydes, with a minimum of 4622.73 ± 314.44 µg/L in PA containing four aldehydes and a maximum concentration of 33852.25 ± 2457.00 µg/L in CAR/PDMS containing five aldehydes. Benzaldehyde and benzeneacetaldehyde were detected in all fibers. PA and CAR/PDMS showed a good detection effect for other aldehydes, such as benzenebutanal and ethylidene-benzeneacetaldehyde. Most ketones were detected in somewhat less amounts than aldehydes in different fibers. There were greater adsorption differences when different fibers were used. Dihydro-5-pentyl-2(3H)-furanone, benzophenone, and 1,1-diphenyl-2-propanone only were detected in PA; acetophenone only was detected in CAR/PDMS, and octan-3-one was only detected in PDMS and DVB/CAR/PDMS. Carbonyl compounds were also common odor components in rice wine. Benzaldehyde and octan-3-one were detected in rice wine [6,9]. Aldehydes and ketones are believed to result from the direct oxidation of their corresponding alcohols and fatty acids, respectively [31]. Other authors suggested that carbonyls result from the degradation of amino acids and sugar [32]. On the basis of the OAV, the most potentially important aroma aldehyde was benzeneacetaldehyde (OAV ranging from 56.7 to 214.47), which imparted a sweet and floral aroma. Benzaldehyde may be the second most important aroma aldehyde, which had a OAV ranging from 10.8 to 93.1 and provided sweet and fruity to the sample. In the detected ketones, octan-3-one, which is known for its fruity odor, had a high OAV in CAR/PDMS (9.6) and DVB/CAR/PDMS (18.1) fiber coatings.

#### 2.2.4. Benzene Derivatives, Hydrocarbons, and Terpenes

Twelve benzene derivatives, 12 hydrocarbons, and two terpenes were detected using four fibers, which showed high number and relative concentration in sample. Nine, seven, ten, and ten benzene derivatives, with subtotal concentrations 1403.20 ± 60.07 μg/L, 2320.13 ± 47.47 μg/L, 4662.34 ± 203.48 μg/L, and 11051.34 ± 850.84 μg/L, respectively, were detected in PA, PDMS, CAR/PDMS, and DVB/CAR/PDMS, respectively. DVB/CAR/PDMS showed a good absorbing effect for benzene derivatives. Two, 12, eight, and nine hydrocarbons, which had a subtotal concentration of 123.17 ± 10.38 μg/L, 6776.38 ± 328.08 μg/L, 3366.62 ± 206.65 μg/L, and 6015.91 ± 397.40 μg/L, respectively, were identified in PA, PDMS, CAR/PDMS, and DVB/CAR/PDMS, respectively. PDMS showed a good absorbing effect for hydrocarbons in number and concentration. Only two terpenes were detected in samples with low concentration. Pinene and limonene were all detected in PDMS. Comparing with rice, abundant benzene derivatives and hydrocarbons were detected in proso millet wine. This could be due to crop differences. Usually, the source of benzene derivatives and hydrocarbons were from the external environment such as water and air [33]. These benzene derivatives and hydrocarbons which were detected in proso millet wine were also detected in proso millet [2]. Thus, these compounds are more likely to come from raw materials. Benzene derivatives were the most important odor component in the sample. Among five odor characteristic benzene derivatives, the OAV of four compounds was greater than 1, especially biphenyl, which had a high OAV in CAR/PDMS and DVB/CAR/PDMS, i.e., OAV reached 254.4 and 355.7. Other benzene derivatives, 1-methyl-naphthalene and 2-methyl-naphthalene, which both had an OAV greater than 1 in each fiber and ranged from 9.7 to 96.2, contributed phenolic aroma to the sample. The OAV of styrene ranged from 10.4 to 25.1 in PDMS, CAR/PDMS, and DVB/CAR/PDMS; styrene generally has a relationship to the sweet and floral characteristics of the wine. Hydrocarbons, which also were detected in the raw material [2], likely have little contribution to the odor of proso millet wine due to odorless feature. However, some hydrocarbons are present in the samples with relatively higher concentration, and may thus play roles in the overall flavor. The PDMS fiber coat was found to have good absorbability for terpenes, which belonged to the unipolar compound. Similar to hydrocarbons and benzene derivatives, terpene could also come from proso millet [2]. Limonene generally relates to citrus aroma [25]; however, the OAV was less than 1 for all fibers, and thus there was little contribution to the odor.

#### 2.2.5. Other Compounds

Seven other compounds, which included three phenols, two acids, and two heterocycles, were detected in the sample. The PA fiber coat had a good absorption efficiency for polar compounds such as phenols and acids. Among of these compounds, phenols could have been formed from lignin degradation of raw materials [34], acids were a product of microbial fermentation [34], and nitrogen-containing compounds could be formed through both non-enzymatic and enzymatic pathways [34]. In all of the other compounds, only benzisothiazole, for which the OAV exceeded 1, was likely to contribute a weak gasoline or rubber aroma to the sample.

Obviously, among the 64 volatile compounds, 26 have been established and their odor threshold and description were published. To some extent, it influenced the comprehensive understanding of odor characteristics of proso wine. However, with the development of flavor chemistry, more volatile compounds odor thresholds and descriptions will be established, which will help us deeply understand aroma quality of proso millet wine.

### 2.3. Principal Component Analysis (PCA)

Principal component analysis (PCA) was used to evaluate 64 volatile compounds and 14 odor-active compounds (OAVs > 1) in proso millet wine using SPME with four fibers.

#### 2.3.1. Volatile Compounds in Proso Millet Wine using SPME with Different Fibers as Indicated by PCA

**Figure 2:**
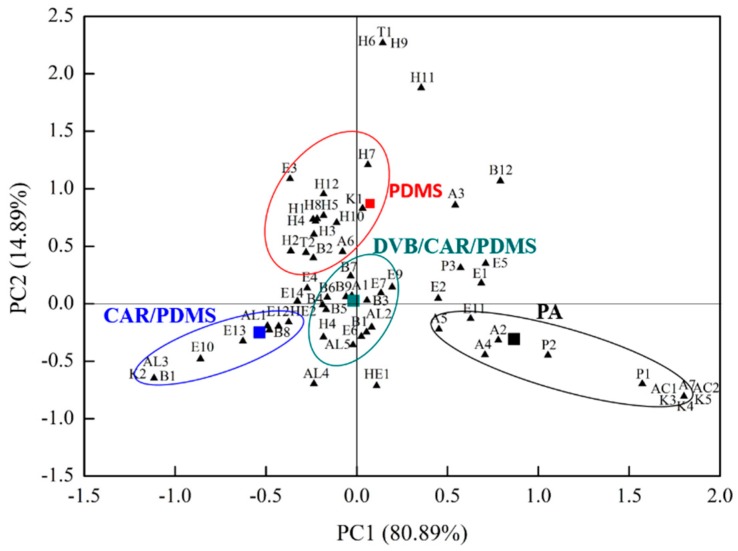
Principal component analysis (PCA) biplot showing relationship between the four fibers and 64 volatile compounds in proso millet wine.

In Figure 2, the scatter plot shows scores for the two first principal components (PC1 and PC2), indicating the volatile compounds distribution differences of in proso millet wine using SPME with four fibers. PC1 and PC2 explained approximately 80.89% and 14.89% of the total variation, respectively (95.78% collectively). PA lay in the positive region of PC1 and the negative region of PC2. Alcohols (A2, A4, A5, and A7), ketones (K3, K4, and K5), acids (AC1 and AC2), phenols (P1 and P2), and esters (E1) are all volatile compounds and were here found to be closely related to PA fibers. Polyacrylate-coated PA showed good absorption for strongly polar compounds [35]. PDMS lay in the positive region of PC1 and PC2. Hydrocarbons (H1, H1, H3, H4, H5, H7, H8, H10, and H12), an ester (E3), an alcohol (A6), and a ketone (K1) were closely correlated with PDMS. PDMS fiber, which is composed of polydimethylsiloxane, is effective for the extraction of nonpolar compounds [35]. CAR/PDMS lay in the negative region of PC1 and PC2, and it is related to esters (E10, E12, and E13), aldehydes (AL1 and AL3), and benzene derivatives (B1 and B8). DVB/CAR/PDMS was located near the original point, where benzene derivatives (B1, B4, B5, B6, B7, B8, and B9), esters (E6, E7, and E9), aldehydes (AL2 and AL3), an alcohol (A1), and a hydrocarbon (H4) were clustered. DVB/CAR/PDMS had a divinylbenzene–Carboxen carbon molecular sieve and a PDMS-fitted coating fibrous layer, which were together more sensitive and selective than PDMS and CAR/PDMS [35].

#### 2.3.2. Odor Active Compounds in Proso Millet Wine Examined using SPME with Different Fibers

**Figure 3:**
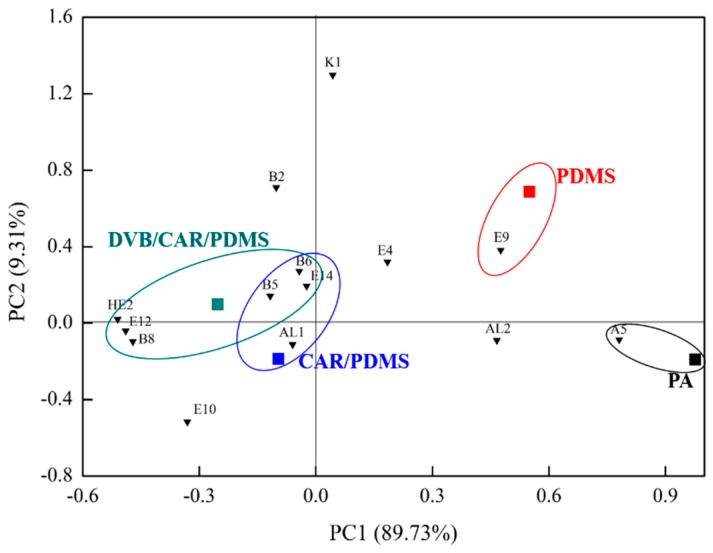
PCA biplot showing relationship between the four fibers and 14 odor-active compounds (OAVs > 1) in proso millet wine.

As shown in Figure 3, the scatter plot for the two first principal components (PC1 and PC2) indicates the differences in proso millet wine using SPME with four fibers. PC1 and PC2 explained approximately 89.73% and 9.31% of the total variation, respectively (99.04% collectively). PA lay in the fourth quadrant of the PCA biplot. The floral and fruity notes of A5 were closely correlated with PA fiber. PDMS lay in the first quadrant of the PCA biplot, and it was found to be related to the fruity and sweet notes of E9. CAR/PDMS lay in the third quadrant of the PCA biplot. The phenolic and medical notes associated with B5 and B6, the floral and fruity notes associated with E14, and the sweet notes associated with AL1 were found to be correlated with CAR/PDMS. DVB/CAR/PDMS lay in the second quadrant of the PCA biplot. The phenolic and medical notes associated with B5, B6, and B7, the floral and fruity notes associated with E12 and E14, and the gasoline note associated with HE2 were found to be correlated with DVB/CAR/PDMS. In addition, the aromas of B5, B6, and E14 were similar to those of CAR/PDMS and DVB/CAR/PDMS.

## 3. Materials and Methods

### 3.1. Proso Millet Wine

Commercialized proso millet wine, which had been pasteurized and put in 500 mL glass bottles was obtained directly from a manufacturer (Zhangjiakou in the North Yellow Wine Co. Ltd., Zhangjiakou, China). The samples were stored in the dark at 4 °C. Alcohol, pH, total acidity, total sugar, non-sugar solids, amino acid nitrogen, and ash (Table 2) analyses were conducted according to the standard methods (AOAC, 1997).

### 3.2. HS-SPME

Four types of SPME fibers with different coatings were purchased from Supelco Inc. (Bellefonte, PA, USA). They were 85 μm polyacrylate (PA), 100 μm polydimethylsiloxane (PDMS), 75 μm Carboxen/polydimethylsiloxane (CAR/PDMS), and 50/30 μm DVB/CAR/PDMS. The fibers used were preconditioned before analysis in the injection port of the gas chromatograph according to the manufacturer’s instructions. An amount of 85 μm of PA was aged for 60 min at 280 °C, and 100 μm of PDMS was aged for 30 min at 250 °C; 75 μm of CAR/PDMS was aged for 60 min at 300 °C; and 50/30 of μm DVB/CAR/PDMS was aged for 60 min at 270 °C.

For the sample, NaCl was placed in a 15 mL vial. Before the SPME fiber was inserted into the vial, the vial was sealed with one Teflon-faced septum and equilibrated for 20 min in a water bath. After that, the fiber was exposed in the headspace of the sealed vial to extract compounds. Preliminary experiments were carried out to evaluate the HS-SPME process by optimizing the main parameters, i.e., sample volume, extraction time, extraction temperature, and NaCl addition. After extraction, the fiber was inserted into the injection port of the gas chromatograph (250 °C) for 5 min to desorb the analytes. The internal standard octan-3-ol solution at 50 mg/L in absolute ethanol was added to the sample under optimal extraction conditions to quantify each compound listed in Table 1. Extraction of each sample was performed in triplicate. After extraction, n-alkanes (C8-C20) were injected under the same conditions to calculate the retention indices.

### 3.3. GC-MS

The gas chromatography-mass spectrometry (GC-MS) procedure used in this study was described by Liu et al 2015 [30]. GC-MS was performed using an HP 5975B quadrupole mass selective detector (Agilent Technologies, Santa Clara, CA, USA). The mass spectral ionization temperature was set to 230°C. The mass spectrometer was operated in the electron impact ionization mode at a voltage of 70 eV. Mass spectra were taken over an m/z range of 30–400. The flow rate of the helium carrier gas on the DB-5 column (30 m × 0.25 mm ID, 0.25 μm film thickness, J&W Scientific, Folsom, CA, USA) was 1 mL/min. The analysis was performed in the splitless mode, and the injector temperature was 250°C. The column was held at 40 °C for 3 min and then increased from 40 °C to 220 °C at a rate of 4 °C/min, held at 220 °C for 2 min, and finally increased to 230 °C at a rate of 8 °C/min and held for 3 min.

### 3.4. Identification of Components

The volatile components were identified by comparing their mass spectra to spectra from MS libraries (NIST 05, WILEY 7.0). The linear retention indexes (RI) of the compounds were calculated using a series of n-alkanes (C_8_-C_20_). Identifications were confirmed by comparing Kovats RI with literature data, or only by MS libraries when literature data were not available.

### 3.5. OAV

The OAV of each volatile compound was calculated from the equation OAV = c/t, where c is the total concentration of the compound concerned in the wine and t its odor threshold value [22,23].

### 3.6. Statistical Analysis

The volatile compounds identified and quantified were listed in the table, which was created in Microsoft Office Excel 2013, also in which are the mean value, standard deviation, and the OAVs. PCA was performed using SAS for Windows Version 8 (SAS Institute, Cary, NC, USA).

## 4. Conclusions

The extraction conditions for the HS-SPME with PA, PDMS, CAR/PDMS, and DVB/CAR/PDM fibers were optimized, and the different SPME fibers had consistent optimal conditions for detection. A total of 64 volatile compounds and 14 aroma-active compounds were identified from the sample using SPME with different fibers. Ethyl benzeneacetate, phenylethyl alcohol, and benzaldehyde, which all had concentrations greater than 1000 μg/L in each fiber coating, were the main volatile compounds in the samples. The OAV results indicated that seven odor-active compounds, i.e., phenylethyl alcohol, styrene, 1-methyl-naphthalene, 2-methyl-naphthalene, benzaldehyde, benzeneacetaldehyde, and 2-methoxy-phenol, were the prominent odor-active compounds that had a high OAV. PCA showed a strong correlation between the 64 volatile compounds and 14 odor-active compounds and four SPME fibers. PA was efficient in the extraction of polar acids and alcohols; PMDS was efficient in the extraction of nonpolar hydrocarbons; and DVB/CAR/PDMS was efficient in the extraction of medium polar esters and benzene derivatives. The distribution of the intensities of odor characteristics with different SPME fibers was also affected.

## Figures and Tables

**Table 1:** Content and OAV of flavor compounds in proso millet wine using SPME with different fibers (n ± RSD).

No^a^	RI (Literature)^b^	Compounds	Content (μg/L)				Threshold (μg/L)^c^	Odor Description^c^			OAV	
			PA	PDMS	CAR/PDMS	DVB/CAR/PDMS			PA	PDMS	CAR/PDMS	DVB/CAR/PDMS
		Esters										
E1	1086(1061)	Ethyl 2-hydroxycaproate	948.32 ± 87.21	505.43 ± 31.31	ND	1101.33 ± 47.89						
E2	1082(1042)	Isoamyl lactate	320.16 ± 23.14	157.00 ± 18.21	120.75 ± 29.38	413.03 ± 62.23						
E3	1099(1107)	Ethyl heptanoate	ND	420.31 ± 62.39	286.32 ± 23.61	ND	14000	Fruity, sweet	ND	0^d^	0	ND
E4	1170(1176)	Ethyl benzoate	116.69 ± 6.06	260.25 ± 12.66	533.69 ± 33.93	404.93 ± 37.60	500	Fruity	0.2	0.5	1.1	0.8
E5	1182(1188)	Diethyl butanedioate	1210.03 ± 74.69	892.01 ± 82.36	ND	1110.34 ± 116.03	100000	Floral	0	0	ND	0
E6	1224(1224)	Ethyl 3-pyridinecarboxylate	63.53 ± 4.87	ND	90.56 ± 5.35	185.35 ± 3.70						
E7	1243(1229)	Ethyl benzeneacetate	1448.89 ± 73.30	1190.98 ± 100.25	1664.30 ± 109.39	2197.40 ± 221.64						
E8	1285(1281)	Diethyl pentanedioate	54.79 ± 9.32	ND	ND	ND						
E9	1343(1355)	Ethyl 3-phenylpropanoate	244.53 ± 22.11	206.19 ± 10.33	216.38 ± 6.40	442.87 ± 28.14	40	Fruity, sweet	6.1	5.2	5.4	11.1
E10	1396(1395)	Ethyl decanoate	ND	ND	219.36 ± 34.90	67.15 ± 1.18	200	Fruity, sweet	ND	ND	1.1	0.3
E11	1449(1454)	Dimethyl phthalate	3882.64 ± 150.31	1046.41 ± 43.97	1072.81 ± 93.54	3347.58 ± 313.74						
E12	1597(1593)	Ethyl dodecanoate	ND	ND	220.65 ± 7.36	374.74 ± 23.11	200	Floral, fat	ND	ND	1.1	1.9
E13	1790(1793)	Ethyl tetradecanoate	ND	ND	360.85 ± 75.06	293.84 ± 65.88	500	Sweet, creamy	ND	ND	0.7	0.6
E14	1995(1936)	Ethyl hexadecanoate	237.95 ± 40.25	513.99 ± 37.87	1534.63 ± 179.03	1543.67 ± 154.71	1000	Fruity, creamy	0.2	0.5	1.5	1.5
		Subtotal	8527.53 ± 27.26	5192.56 ± 292.54	6320.30 ± 452.59	11482.29 ± 550.77						
		Alcohols										
A1	850(865)	2-furanmethanol	ND	ND	ND	513.42 ± 13.07	1500	Sweet burning	ND	ND	ND	0.3
A2	865(887)	Hexan-1-ol	1401.96 ± 68.53	ND	ND	1779.04 ± 280.87						
A3	1033(1033)	2-ethyl-hexan-1-ol	238.33 ± 35.77	413.28 ± 32.00	ND	237.18 ± 27.26	270000	Mild, oily	0	0	ND	0
A4	1051(1039)	Benzyl alcohol	144.23 ± 47.19	ND	39.63 ± 7.91	117.38 ± 17.94	159000	Floral, sweet	0	ND	0	0
A5	1116(1120)	Phenylethyl alcohol	12826.54 ± 1430.61	2996.79 ± 303.73	7192.58 ± 804.06	10648.48 ± 1547.25	900	Floral, fruity	14.3	3.3	8.0	11.8
A6	1174(1177)	4-methyl-1-(1-methylethyl)-3-cyclohexen-1-ol	51.89 ± 7.11	136.59 ± 17.52	128.36 ± 7.84	108.14 ± 34.28						
A7	1189(1179)	4-trimethyl-3-cyclohexene-1-methanol	319.62 ± 25.61	ND	ND	ND						
		Subtotal	14982.57 ± 1464.48	3546.66 ± 339.05	7360.57 ± 800.93	13403.63 ± 1335.52						
		Aldehydes										
AL1	962(968)	Benzaldehyde	3778.48 ± 273.55	5031.70 ± 327.21	32579.19 ± 2487.86	18258.22 ± 1762.50	350	Sweet, fruity	10.8	14.4	93.1	52.2
AL2	1057(1046)	Benzeneacetaldehyde	636.55 ± 19.98	226.95 ± 23.05	857.89 ± 51.04	747.47 ± 129.41	4	Sweet, floral	159.1	56.7	214.5	186.9
AL3	1148(1152)	2-phenyl-propenal	ND	ND	19.43 ± 3.41	ND						
AL4	1280(NL) ^a^	Benzenebutanal	78.88 ± 7.76	ND	182.65 ± 4.40	ND						
AL5	1283(1273)	Ethylidene-benzeneacetaldehyde	128.83 ± 19.08	ND	213.09 ± 13.86	276.63 ± 8.34						
		Subtotal	4622.73 ± 314.44	5258.66 ± 304.79	33852.25 ± 2457.00	19282.32 ± 1833.79						
		Ketones										
K1	980(984)	Octan-3-one	ND	545.81 ± 37.02	ND	1033.00 ± 137.94	57	Fruity	ND	9.6	ND	18.1
K2	1072(1065)	Acetophenone	ND	ND	33.51 ± 2.91	ND	170	Sweet, pungent	ND	ND	0.2	ND
K3	1372(1363)	Dihydro-5-pentyl-2(3H)-furanone	211.18 ± 19.18	ND	ND	ND						
K4	1612(1644)	Benzophenone	64.00 ± 4.99	ND	ND	ND						
K5	1737(NL)	1,1-diphenyl-2-propanone	85.41 ± 4.27	ND	ND	ND						
		Subtotal	360.59 ± 17.94	545.81 ± 37.02	33.51 ± 2.91	1033.00 ± 137.94						
		Benzene derivatives										
B1	861(855)	Xylene	ND	ND	578.45 ± 41.24	ND	2300	Plastic	ND	ND	0.3	ND
B2	874(868)	Styrene	ND	829.06 ± 108.90	848.14 ± 34.46	2006.43 ± 123.63	80	Sweet, floral	ND	10.4	10.6	25.1
B3	897(NL)	Methoxy-phenyl-oxime	156.12 ± 14.98	ND	ND	3283.89 ± 597.99						
B4	1179(1179)	Naphthalene	131.13 ± 8.05	154.04 ± 14.46	520.44 ± 56.05	1062.92 ± 51.90						
B5	1296(1305)	1-methyl-naphthalene	300.33 ± 14.83	251.58 ± 14.77	994.20 ± 42.32	1923.45 ± 218.17	20	Phenolic	15.0	12.6	49.7	96.2
B6	1311(1284)	2-methyl-naphthalene	193.45 ± 6.25	278.52 ± 5.40	661.96 ± 38.97	1222.16 ± 45.89	20	Phenolic	9.7	13.9	33.1	61.1
B7	1378(NL)	(2,2-diethoxyethyl)-benzene	353.07 ± 20.86	563.58 ± 61.83	697.21 ± 56.38	911.79 ± 55.43						
B8	1378(1351)	Biphenyl	ND	ND	127.197.25	177.87 ± 21.95	0.5	Medicine	ND	ND	254.4	355.7
B9	1405(1426)	2,6-dimethyl-naphthalene	25.87 ± 0.02	26.94 ± 1.32	53.15 ± 2.63	81.72 ± 12.58						
B10	1418(1442)	2,3-dimethyl-naphthalene	31.16 ± 4.27	ND	88.66 ± 7.77	132.70 ± 22.16						
B11	1584(1556)	Fluorene	73.60 ± 8.83	ND	92.92 ± 19.44	248.40 ± 49.44						
B12	1753(1794)	Phenanthrene	138.48 ± 14.45	216.40 ± 25.94	ND	ND						
		Subtotal	1403.20 ± 60.07	2320.13 ± 47.47	4662.34 ± 203.48	11051.34 ± 850.84						
		Hydrocarbons										
H1	1200(1200)	Dodecane	ND	797.36 ± 29.44	530.01 ± 76.52	739.63 ± 58.49						
H2	1300(1300)	Tridecane	ND	53.24 ± 11.94	64.41 ± 4.53	64.18 ± 13.56						
H3	1389(1391)	Tetradec-1-ene	ND	163.23 ± 5.86	122.77 ± 13.87	221.17 ± 17.62						
H4	1400(1400)	Tetradecane	ND	602.56 ± 57.93	390.77 ± 54.42	622.17 ± 167.76						
H5	1500(1500)	Pentadecane	ND	1377.58 ± 116.92	730.28 ± 76.17	1485.64 ± 42.45						
H6	1564(1564)	2-methyl-pentadecane	ND	281.76 ± 21.63	ND	ND						
H7	1593(1591)	Hexadec-1-ene	ND	286.14 ± 11.02	ND	266.65 ± 33.29						
H8	1600(1600)	Hexadecane	ND	1654.72 ± 103.74	1022.11 ± 75.78	1667.06 ± 121.25						
H9	1627(1629)	2,6,10-trimethyl-pentadecane	ND	355.01 ± 10.14	ND	ND						
H19	1700(1700)	Heptadecane	86.72 ± 9.33	812.34 ± 32.73	440.78 ± 77.50	855.65 ± 90.69						
H11	1795(1794)	Octadec-1-ene	36.45 ± 4.40	249.53 ± 7.79	ND	ND						
H12	1800(1800)	Octadecane	ND	142.91 ± 10.92	65.50 ± 9.46	93.77 ± 7.12						
		Subtotal	123.17 ± 10.38	6776.38 ± 328.08	3366.62 ± 206.65	6015.91 ± 397.40						
		Terpene										
T1	948(939)	Pinene	ND	386.72 ± 38.41	ND	ND						
T2	1033(1041)	Limonene	ND	83.37 ± 11.01	87.34 ± 10.58	149.60 ± 7.50	250	Limonene	ND	0.3	0.4	0.6
		Subtotal	0	470.09 ± 47.23	87.34 ± 10.58	149.60 ± 7.50						
		Phenols										
P1	986(980)	Phenol	580.50 ± 46.18	ND	ND	83.53 ± 8.69						
P2	1277(NL)	2-(1,1-dimethylethyl)-6-methyl-phenol	173.04 ± 19.26	ND	ND	120.09 ± 20.25						
P3	1519(1512)	2,4-bis(1,1-dimethylethyl)-phenol	2788.87 ± 191.24	2226.50 ± 308.71	824.54 ± 22.62	1815.72 ± 107.49						
		Subtotal	3542.41 ± 205.79	2226.50 ± 308.71	824.54 ± 22.62	2019.33 ± 106.73						
		Acids										
AC1	1072(1013)	Heptanoic acid	245.04 ± 26.27	ND	ND	ND	500	Fatty, rancid	0.5	ND	ND	ND
AC2	1172(1182)	Octanoic acid	79.22 ± 3.36	ND	ND	ND	500	Fatty, rancid	0.2	ND	ND	ND
		Subtotal	324.26 ± 27.04	0	0	0						
		Heterocycles										
HE1	1082(1096)	Tetramethyl-pyrazine	180.77 ± 20.86	ND	249.65 ± 41.82	ND	1000	Floral, fruity	0.2	ND	0.3	ND
HE2	1218(1205)	Benzisothiazole	ND	ND	107.74 ± 5.27	229.82 ± 27.54	80	Gasoline	ND	ND	1.4	2.9
		Subtotal	180.77 ± 20.86	0	357.39 ± 36.66	229.82 ± 27.54						
		Total	34067.24 ± 2037.95	26336.79 ± 763.85	56864.85 ± 2010.45	64667.24 ± 2002.81						

RSD: Relative standard deviation; RI: Retention indices on a DB-5 column; OAV: Odor activity value = concentration/odor threshold; NL: No literature data; ND: No detection; PA: polyacrylate; PDMS: polydimethylsiloxane; CAR: Carboxen; DVB, divinylbenzene; ^a^ Compounds’ No was composed of compounds’ acronym + number, E, A, AL, K, B, H, T, P, AC, and HE represented ester, alcohol, aldehyde, ketone, benzene derivative, hydrocarbon, terpene, phenol acid heterocycle, respectively; ^b^ The literature data were reported in [2], [10], [14] and https://www.nist.gov/; ^c^ Threshold and odor description were reported in [23] [24] and [25]; ^d^ 0 in OAV expressed OAV <0.01.

**Table 2:** General composition of the foxtail millet sake (n ± RSD).

Alcohol (% *v*/*v*)	pH	Total Acidity (g/L as Lactic Acid)	Total Sugar (g/L as Glucose)	Non-Sugar Solidity (g/L)	Amino Acid Nitrogen (g/L)	Ash (g/L)
13.54 ± 0.99	3.19 ± 0.14	3.25 ± 0.18	10.06 ± 1.28	10.76 ± 1.11	0.32 ± 0.11	0.98 ± 0.11

## References

[1] Gulati P., Weier S.A., Santra D., Subbiah J., Rose D.J. (2016). Effects of feed moisture and extruder screw speed and temperature on physical characteristics and antioxidant activity of extruded proso millet (*Panicum miliaceum*) flour. Int. J Food Sci. Tech..

[2] Liu J., Tang X., Liu Y., Zhang Y., Zhao W., Li S. (2013). Analysis of volatile aroma compounds from congenetic proso millet (*Panicum miliaceum* L.). J. Food Agric. Environ..

[3] Romero H.M., Santra D., Rose D., Zhang Y. (2017). Dough rheological properties and texture of gluten-free pasta based on proso millet flour. J. Cereal Sci..

[4] Srivastava S., Thathola A., Batra A. (2001). Development and nutritional evaluation of proso millet-based convenience mix for infants and children. J. Food Sci. Tech. Mys..

[5] Schoenlechner R., Szatmari M., Bagdi A., Toemoeskoezi S. (2013). Optimisation of bread quality produced from wheat and proso millet (*Panicum miliaceum* L.) by adding emulsifiers, transglutaminase and xylanase. LWT-Food Sci. Technol..

[6] Wang D., Jin B., Xu Y., Zhao G. (2013). Study on flavor sensory characteristics and the construction of flavor wheel for Chinese rice wine. Food Sci..

[7] Chen S., Xu Y., Qian M.C. (2013). Aroma characterization of Chinese rice wine by gas chromatography-olfactometry, chemical quantitative analysis, and aroma reconstitution. J. Agr. Food Chem..

[8] Kaneko S., Kumazawa K. (2015). Aroma compounds in Japanese sweet rice wine (Mirin) screened by aroma extract dilution analysis (AEDA). Biosci. Biotech. Bioc..

[9] Luo T., Fan W.L., Xu Y. (2008). Characterization of volatile and semi-volatile compounds in Chinese rice wines by headspace solid phase microextraction followed by gas chromatography-mass spectrometry. J. I. Brew..

[10] Fan W., Qian M.C. (2005). Headspace solid phase microextraction and gas chromatography-olfactometry dilution analysis of young and aged Chinese “Yanghe Daqu” liquors. J. Agr. Food Chem..

[11] Johnson S.R., Soprano S.E., Wickham L.M., Fitzgerald N., Edwards J.C. (2017). Nuclear magnetic resonance and headspace solid-phase microextraction gas chromatography as complementary methods for the analysis of beer samples. Beverages.

[12] Xu Y., Fan W., Qian M.C. (2007). Characterization of aroma compounds in apple cider using solvent- assisted flavor evaporation and headspace solid-phase microextraction. J. Agr. Food Chem..

[13] Cai L., Rice S., Koziel J., Dharmadhikari M. (2017). Development of an automated method for selected aromas of red wines from cold-hardy grapes using solid-phase microextraction and gas chromatography-mass spectrometry-olfactometry. Separations.

[14] Kataoka H., Lord H.L., Pawliszyn J. (2000). Applications of solid-phase microextraction in food analysis. J. Chromatogr. A..

[15] Hansen A.S., Frandsen H.L., Fromberg A. (2016). Authenticity of raspberry flavor in food products using SPME-chiral-GC-MS. Food Sci. Nutr..

[16] Gholivand M.B., Piryaei M., Abolghasemi M.M. (2013). Analysis of volatile oil composition of citrus aurantium L. by microwave-assisted extraction coupled to headspace solid-phase microextraction with nanoporous based fibers. J. Sep. Sci..

[17] Lorenzo J.M. (2014). Influence of the type of fiber coating and extraction time on foal dry-cured loin volatile compounds extracted by solid-phase microextraction (SPME). Meat Sci..

[18] Zhou J., Han Y., Zhuang H., Feng T., Xu B. (2015). Influence of the type of extraction conditions and fiber coating on the meat of sauced duck neck volatile compounds extracted by solid-phase microextraction (SPME). Food Anal. Method..

[19] Liu Y., Miao Z., Guan W., Sun B. (2012). Analysis of organic volatile flavor compounds in fermented stinky Tofu using SPME with different fiber coatings. Molecules..

[20] Vilanova M., Martinez C. (2007). First study of determination of aromatic compounds of red wine from Vitis vinifera CV. Castañal grown in Galicia (NW Spain). Eur. Food Res. Technol..

[21] Silva C.E., Costa W.F., Minguzzi S., Silva R.C., Simionatto E. (2013). Assessment of volatile chemical composition of the essential oil of Jatropha ribifolia (Pohl) baill by HS-SPME-GC-MS using different fibers. J. Anal. Methods Chem..

[22] Câmara J.S., Arminda Alves M., Marques J.C. (2006). Development of headspace solid-phase microextraction gas chromatography-mass spectrometry methodology for analysis of terpenoids in Madeira wines. Anal. Chim. Acta.

[23] Jiang B., Zhang Z. (2010). Volatile compounds of young wines from cabernet sauvignon, cabernet gernischet and chardonnay varieties grown in the loess plateau region of China. Molecules.

[24] Burdock G.A. (2009). Fenaroli’s Handbook of Flavor Ingredients Sixth Edition.

[25] Kummer R., Fachiniqueiroz F.C., Estevãosilva C.F., Grespan R., Silva E.L., Bersaniamado C.A., Cuman R.K.N. (2013). Evaluation of anti-inflammatory activity of Citrus latifolia Tanaka essential oil and limonene in experimental mouse models. Evid. Based Complement. Alternat. Med..

[26] Yan Z., He M., Chen B., Gui B., Wang C., Hu B. (2017). Magnetic covalent triazine framework for rapid extraction of phthalate esters in plastic packaging materials followed by gas chromatography-flame ionization detection. J. Chromatogr. A..

[27] Fan W.M., Qian C. (2006). Identification of aroma compounds in Chinese ‘Yanghe Daqu’ liquor by normal phase chromatography fractionation followed by gas chromatography-olfactometry. Flavour Frag. J..

[28] Wondra M., Berovic M. (2001). Analyses of aroma components of Chardonnay wine fermented by different yeast strains. Food Technol. Biotech..

[29] Fukuda K., Watanabe M., Asano K., Hiroyuki U., Shigenori O. (1990). Breeding of brewing yeast producing a large amount of β-phenylethyl alcohol and β-phenylethyl cetate. Agr. Boil. Chem..

[30] Liu J., Zhao W., Li S., Zhang A., Zhang Y. (2015). Determination of volatile compounds in foxtail millet sake using headspace solid-phase microextraction and gas-chromatography mass spectrometry. J. Chem..

[31] Campo E., Ferreira V., Escudero A., Marqués J.C., Cacho J. (2006). Quantitative gas chromatography- olfactometry and chemical quantitative study of the aroma of four Madeira wines. Anal. Chim. Acta.

[32] Camara J.S., Marques J.C., Alves M.A., Ferreira A.C.S. (2004). 3-Hydroxy-4,5-dimethyl-2(5H)- furanone levels in fortified Madeira wines: relationship to sugar content. J. Agr. Food Chem..

[33] Weldegergisa B.T., Villiers A., McNeishb C., Seethapathy S., Mostafa A., Góreckib T., Croucha A.M. (2011). Characterisation of volatile components of Pinotage wines using comprehensive two-dimensional gas chromatography coupled to time-of-flight mass spectrometry (GC×GC-TOF-MS). Food Chem..

[34] Mo X., Fan W., Xu Y. (2009). Changes in volatile compounds of Chinese rice wine wheat qu during fermentation and storage. J. I. Breing..

[35] Merkle S., Kleeberg K.K., Fritsche J. (2015). Recent developments and applications of solid phase microextraction (SPME) in food and environmental analysis—a review. Chromatogr..

